# Identification of Yeasts and Bacteria Isolated From Iranian Kefir Drink

**DOI:** 10.5812/jjm.10707

**Published:** 2014-02-01

**Authors:** Seyyed Ali Asghar Sefidgar, Samane Gharekhani, Maryam Ghasempour

**Affiliations:** 1 Department of Mycology and Parasitology, School of Medicine, Babol University of Medical Sciences, Babol, IR Iran; 2 Department of Pediatric, School of Dentistry, Babol University of Medical Sciences, Babol, IR Iran

**Keywords:** Bacteria, Kefir, Yeast

*Dear Editor,*

Kefir drink is a popular natural probiotic beverage in the Middle East. It is the fermentation product of a variety of probiotic microorganisms- live microorganisms added to the diet in sufficient quantities to improve the health condition- present in the kefir grain of the milk (1). Different kefir grains may have various microbial complexes (2). Generally, knowing the microbial characterizations of probiotic products is needed for the safety confirmation. On the other hand, the beneficial effects of probiotics vary from strain to strain and condition to condition (3). This study was performed to isolate the kefir microorganisms by culturing and biochemical tests.

The kefir grains were obtained from a family in the North of Iran. Five percent of this starter was added to the cooled pasteurized milk at 20^o^C (3% fat, Kale dairy Co., Iran) and incubated at 30^o^C for 48 hours. The produced beverage was filtered to remove the kefir grains and its pH was measured using the pH indicator paper (Arak Chemical Co., Iran). To identify the yeasts, One mL of homemade kefir drink was diluted to 10^-6^, 0.1 mL of this solution was inoculated on Man Rogosa Sharpe (MRS) broth medium (Himedia, India) and incubated at 37^o^C for 48 hours. Then, one loop was inoculated on MRS agar medium at the same condition. Regarding the size and morphology, the different colonies were analyzed by the Gram staining (Labtron, Iran), Germ tube test, Urea hydrolysis (Himedia, India), Cycloheximide resistance (AppliChem GmbH, Germany) and fermentation of sugars (Sigma- Aldrich, USA).

To isolate the Lactic acid Bacteria (LAB)**, **one-tenth mL of the same dilution was added to MRS broth medium incubated at anaerobic conditions (100% CO2). Detecting the LAB was done by the Gram staining (Labtron, Iran), the Catalase (Merck, Germany) and Oxidase (Himedia, India) tests, metabolization of Esculin on Bile Esculin agar medium (Himedia, India), growth in the presence of 0.5 salt (Himedia, India), SH2-indole-motility (Himedia, India) test and fermentation of sugars according to Bergey’s Manual (4).

The pH determined 4.5, at room temperature. The low pH of prepared drink was similar to the result of Tietze HW’s study (5). Producing the lactic acid, ethanol, CO_2_, and other volatile compounds by the microbial population in kefir grain were a best reasons for low pH of this beverage, which makes the environment suitable for aciduric and acidogenic bacteria and yeasts (2). By culturing methods and biochemical tests, *Saccharomyces cerevisiae* and *Lactobacillus casei subsp. pseudo plantarum* were detected. Biochemical characterizations of these microorganisms have been illustrated in Table 1. 

**Table 1.** Biochemical Characterizations of *S. cerevisiae *and* L. casei subsp. pseudo plantarum *

Biochemical Test	*S. cerevisiae*	*L. casei subsp. pseudo plantarum*
**Gram staining**	+	+
**Germ tube test**	-	
**Urea hydrolysis**	-	
**Resistance to Cycloheximide **	-	
**Catalase**		-
**Oxidase**		-
**Metabolization of Esculin**		+
**Motility**		-
**SH**_**2**_		-
**Growth on the presence of 0.5% salt**		+
**Sugar fermentation**		
Lactose	-	+
Sucrose	+	+
Dextrose	+	-
Galactose	+	+
Trehalose	+	
Maltose	+	+
Mannitol		+
Mannose		+
Arabinose		-
Melibiose		-
Xylose		-
Sorbitol		+
Raffinose	-	-

These results were in agreement with the previous studies (6, 7). It has been reported that yeasts, predominantly Lactose-negative species including* S. cerevisiae *comprised less than 20% of the total isolated microflora in the kefir grains (7). However, the presence of yeasts in some food may have a toxic effect on some patients. Antibody against *S. cerevisiae *was found in 60-70% of patients with Crohn’s disease and 10-15% of those with ulcerative colitis. So consumption of dietary products including the yeast should be restricted for these patients (8).

On the other hand, *L. casei subsp. pseudo plantarum *is one of the best documented probiotics to control the gastrointestinal diseases (9). The production of low molecular weight antimicrobial substances with a broad spectrum activity has been shown by this microorganism (10). In conclusion, the Iranian kefir drink with the revealed microbial profile may be administered in many cases but some cautions should be considered for some patients.

## References

[1] Otles S, Cagindi O (2003). Kefir: A Probiotic Dairy-Composition, Nutritional and Therapeutic Aspects.. Pak J Nutr..

[2] Magalhães KT, Pereira GVDM, Campos CR, Dragone G, Schwan RF (2011). Brazilian kefir: structure, microbial communities and chemical composition.. Brazil J Microbiol..

[3] Sanders ME, Akkermans LM, Haller D, Hammerman C, Heimbach J, Hormannsperger G (2010). Safety assessment of probiotics for human use.. Gut Microbes..

[4] Holt JG, Krieg NR, Sneath PHA, Staley JT, Williams ST (1994). Bergey's manual of determinative bacteriology..

[5] Tietze HW (1996). Kefir: For Pleasure and Wellbeing.:.

[6] Motaghi M, Mazaheri M, Moazami N, Farkhondeh A, Fooladi MH, Goltapeh EM (1997). Short Communication: Kefir production in Iran.. World J Microbiol Biotechnol..

[7] Simova E, Beshkova D, Angelov A, Hristozova T, Frengova G, Spasov Z (2002). Lactic acid bacteria and yeasts in kefir grains and kefir made from them.. J Industrial Microbiol Biotechnol..

[8] Walker LJ, Aldhous MC, Drummond HE, Smith BR, Nimmo ER, Arnott ID (2004). Anti-Saccharomyces cerevisiae antibodies (ASCA) in Crohn's disease are associated with disease severity but not NOD2/CARD15 mutations.. Clin Exp Immunol..

[9] Remacle C, Reusens B (2004). Functional foods, ageing and degenerative disease..

[10] Mollendorff V, Wilhelm J. . (2008). Characterization of bacteriocins produced by lactic acid bacteria from fermented beverages and optimization of starter cultures.. :.

